# Comparison of Analgesic Efficacy of Hyperbaric Levobupivacaine With or Without Clonidine in Patients Undergoing Intramedullary Nailing for Fracture Femur Under Subarachnoid Block: A Randomised Controlled Trial

**DOI:** 10.7759/cureus.99961

**Published:** 2025-12-23

**Authors:** Srishti Chandra, Poonam Rani, Rajesh S Rautela, Rashmi Salhotra

**Affiliations:** 1 Anaesthesiology, Critical Care and Pain Medicine, University College of Medical Sciences and Guru Teg Bahadur (GTB) hospital, New Delhi, IND; 2 Anaesthesiology, Critical Care and Pain Medicine, University College of medical sciences and Guru Teg Bahadur (GTB) Hospital, New Delhi, IND; 3 Anaesthesiology, Critical Care and Pain Medicine, University College of Medical Sciences and Guru Teg Bahadur (GTB) Hospital, New Delhi, IND; 4 Anaesthesia, University College of Medical Sciences and Guru Teg Bahadur (GTB) Hospital, New Delhi, IND

**Keywords:** femur shaft fracture, hyperbaric levobupivacaine, intra-medullary nail, intrathecal clonidine, post-operative analgesia

## Abstract

Background

Subarachnoid block is the most widely used technique for lower limb surgeries because of its cost-effectiveness, good muscle relaxation, reliability, prolonged analgesia and early ambulation. Clonidine as an adjuvant for subarachnoid block may improve analgesia. So, in this study, we propose to evaluate the analgesic efficacy of hyperbaric levobupivacaine with or without clonidine in patients undergoing surgery for femoral fractures. Secondarily, we also compared sensory and motor block characteristics and noted side effects.

Methods

Subarachnoid block was given in sitting position under all aseptic precautions at the level of L_2_-L_3_ or L_3_-L_4_ intervertebral space as per the standard protocol. Intrathecal drug was given according to the group allotted as follows: Group-LC: 15 mg of 0.5% hyperbaric levobupivacaine (3 ml) + 15 mcg of clonidine (0.1 ml); Group-LN: 15 mg of 0.5% hyperbaric levobupivacaine (3 ml) + normal saline (0.1 ml)

The total volume of intrathecal drug was 3.1 ml in both groups.

Block characteristics were assessed using loss of pinprick sensation for sensory block and Modified Bromage Scale (MBS) for motor block. Hemodynamic variables were also recorded.

Results

The demographic profiles of both groups were comparable to each other with respect to gender, American Society of Anesthesiologists (ASA) grade, weight and height of patients. The duration of effective analgesia was 166.67 ±31.72 min in group LC and 98.80 ±8.99 min in group LN (p <0.001). The difference was statistically significant. The mean time of onset of sensory block was 6.53 ±1.60 min and 4.00 ±1.69 min (p =0.001); the mean time to achieve a maximum height of the sensory block was 11.53 ±3.72 min and 6.67 ±1.45 min (p <0.001) in groups LC and LN, respectively and were statistically significant. The median maximum block height was comparable in both groups. The maximum motor block achieved and motor block at the time of rescue analgesia were comparable in both groups. Intraoperative and postoperative hemodynamic parameters (heart rate (HR), systolic blood pressure (SBP) and diastolic blood pressure (DBP)) were comparable among the groups LC and LN, except in DBP at 90 mins, where in group LC the mean was 77.00 ±6.32 min and in group LN it was 81.73 ±6.25 min, which was statistically significant. Sedation scores were comparable in both groups intraoperatively and postoperatively. None of the patients had any sedation in either of the groups, and all the patients were easily arousable in group LC and group LN, respectively. No major side effects were observed in either of the groups except one patient (6.7%) in group LC and one patient (6.7%) in group LN developed nausea, and five patients (33.3%) in group LC and three patients (20.0%) in group LN had shivering, which was managed promptly.

Conclusion

Addition of 15 mcg of clonidine to 3 mL (15 mg) of 0.5% hyperbaric levobupivacaine provides a longer duration of effective analgesia with minimal side effects.

## Introduction

Subarachnoid block is a widely used anaesthetic technique for lower limb surgeries. It is safe and effective, with fewer side effects or other complications. It provides reliable anaesthesia, good muscle relaxation and intraoperative and postoperative analgesia without loss of consciousness. It has also been known to decrease the intraoperative blood loss and perioperative risk of cardiac ischaemia. Thus, making it a better option for lower limb surgeries [1, 2].

Various local anaesthetics like bupivacaine, ropivacaine, and levobupivacaine are used for subarachnoid block [3]. Levobupivacaine is an amino-acid local anaesthetic drug that has emerged as a safer alternative to racemic bupivacaine.

To improve the quality of blockade, increase the duration of analgesia, and reduce the required dose of local anaesthetics, which in turn decreases the incidence of side effects caused by high dosages of LA’s various adjuvants are commonly used. However, in clinical practice, the more commonly used additives are opioids and alpha-2 adrenergic agonists.

Clonidine is a selective partial ɑ2 receptor agonist. Neuraxial clonidine is an effective analgesic for chronic cancer and non-cancer pain, as well as for postoperative pain. It causes 30% prolongation of the motor and sensory blocks of LAs. It is commonly administered in doses ranging from 10 to 50 micrograms for subarachnoid block [4-6].

Clonidine has previously been used as an intrathecal adjuvant. Its safety has been studied as an additive to isobaric levobupivacaine. However, at the time of designing this study, there were no studies on its efficacy and safety when used with hyperbaric levobupivacaine. Thus, this study was planned.

Objectives

Primary

To evaluate the analgesic efficacy of hyperbaric levobupivacaine with or without clonidine in patients undergoing intramedullary nailing for femoral fracture under subarachnoid block.

Secondary

Sensory and motor block characteristics (Onset of sensory block, maximum height of sensory block, time to achieve a maximum height of sensory block, maximum motor block achieved).

Hemodynamic changes, Sedation score and ancillary observations like pain score in the postoperative period, side effects during intraoperative or postoperative period were also noted.

## Materials and methods

This randomized, double-blind study was undertaken in a tertiary care hospital in Delhi from 2022 to 2024 after taking approval from the Institutional Ethics Committee - Human Research (IECHR-2022-55-77). The study was registered with Clinical Trials Registry India (ctri.nic.in) with registration number CTRI/2022/12/048579 before enrolling the patients. Written informed consent was taken from each patient involved in this study. Thirty patients were randomly allocated into two groups (n =15 each): Group LC and group LN. Group-LC patients: 15 mg of 0.5% hyperbaric levobupivacaine + 15 µg of clonidine (0.1 ml). Group-LN patients: 15 mg of 0.5% hyperbaric levobupivacaine + normal saline (0.1 ml).

Randomization was done using a computer-generated random number table. Concealment of randomization was done using serially numbered sealed opaque envelopes.

The primary objective was the measurement of the duration of effective analgesia of levobupivacaine with or without clonidine, and the secondary objectives were sensory and motor block characteristics (Onset of sensory block, maximum height of sensory block, time to achieve a maximum height of sensory block, maximum motor block achieved, hemodynamic changes, sedation score in both the groups and ancillary observations like pain score in the postoperative period, side effects during intraoperative or postoperative period).

Inclusion criteria

Thirty patients belonging to ASA physical status I/II, of all genders between 18 and 65 years of age and height 150-180 cm, about to undergo surgery for a femoral fracture requiring an intramedullary nail under a subarachnoid block, were included in the study.

Exclusion criteria

Patients with contraindication to subarachnoid block for any reason, History of allergy to any drug being used in the study, pregnant patients, drug addicts and chronic alcoholics, patients who are on antiarrhythmic drugs or known cases of hepatic and renal disease were excluded from the study.

Sample size was calculated based on a previous study done by Krishna et al., where the duration of effective analgesia was found to be 251.72±31.62 min with levobupivacaine alone and 294.68±43.33 min with levobupivacaine and 15 mcg clonidine [7]. To estimate the mean difference at ɑ=5% and power =80%, a sample of 13 cases was required in each group. To account for the failure and dropout rate of 10%, we proposed to include 15 patients each. So, a total of 30 patients were included.

The eligible patients were informed about the procedure and the type of drug being used, along with its side effects and uses. The concept of the visual analogue scale (VAS) for the assessment of pain was also explained to the patients. Written informed consent was taken from the participants. Anxiolysis and aspiration prophylaxis were given the night before surgery and on the morning of surgery.

Baseline hemodynamic variables were recorded. Intravenous access with an 18-G or 16-G cannula was secured, and preloading was done with Ringer’s lactate by 10 ml/kg within 30 min. Study drugs were prepared by another anaesthesiologist not involved in the study to ensure blinding. The total volume of intrathecal drug was the same, 3.1 ml, in both groups. All observations were made by the observer who was unaware of the intrathecal drug. Both the patient and the observer were blinded to group allocation.

Subarachnoid block (SAB) was given in a sitting position via midline approach under all aseptic precautions, using a 25 G Quincke’s spinal needle at L2-L3 or L3-L4 intervertebral space. The assessment of sensory block was done by the loss of pinprick sensation.

Outcome Measures

1) Duration of effective analgesia was calculated from the time of intrathecal injection up to the time of the patient’s first complaint of pain or when VAS ≥3, whichever was earlier; 2) Sensory block characteristics: Onset: Time taken from the intrathecal drug administration to block the sensory stimuli at the T10 dermatome. Maximum height of sensory block: The block height achieved in three consecutive readings. Time to achieve maximum height of sensory block: Time from intrathecal injection till maximum height. Sensory level, heart rate, blood pressure, and sedation (5-point University of Michigan Sedation Scale) were assessed every 2 min for the first 10 min and then every 5 min till 30 min, every 15 min till the end of 1 hour, and every 30 min thereafter till the end of surgery. Failure: Non-achievement of the sensory level of at least T10 dermatome within 20 min of administration of an intrathecal drug. In such cases, general anaesthesia (GA) was induced according to standard protocol; 3) Motor block parameters: Motor block was assessed at 0, 2, 4, 6, 8, 10, 15 & 20 minutes after giving subarachnoid block and at the time of rescue analgesia using Modified Bromage Scale (MBS) in the normal limb. The maximum motor block was labelled as the maximum motor blockade achieved within the first 20 min; 4) Hemodynamic parameters: Hypotension was defined as 20% decrease in SBP from the baseline or when SBP is <90 mm Hg. It was treated with fluids and vasopressors (Inj. Mephentermine 6 mg IV, as required).

Bradycardia was defined as HR less than 50/min and was treated with 0.6 mg of Atropine IV; 5) Sedation score: Sedation was assessed using the 5-point University of Michigan Sedation Scale at all the time points mentioned under hemodynamic parameter monitoring and was recorded; 6) Ancillary observations: a) Pain score: Pain was assessed by using a 0-10 cm VAS b) Side effects like nausea, vomiting, bradycardia, hypotension, pruritis, urinary retention, etc., during the intraoperative or postoperative period were recorded and treated.

On completion of the surgery, patients were shifted to the postoperative room and monitoring was continued every 30 minutes until the patient complained of pain. Rescue analgesia in the form of Inj. Paracetamol 1 g was given when the patient complained of pain or VAS≥3. This was the endpoint of the study.

Failure

Non-achievement of sensory level of at least T10 dermatome within 20 min of administration of intrathecal drug. In such cases, GA was induced according to standard protocol.

Statistics

Statistical analysis was carried out in SPSS, software version 20.0. One-time measured quantitative parameters were compared using the unpaired t-test/Mann-Whitney’s test. Repeated measures ANOVA followed by Tukey’s test was used to study within and between the group differences. Qualitative data were analysed using the Chi-square test or Fisher’s exact test, whichever was applicable. A p-value < 0.05 was considered significant.

## Results

A total of 35 patients were assessed for eligibility. Out of these, three patients did not meet the inclusion criteria, and another two patients did not give consent to participate in the study.

**Figure 1:**
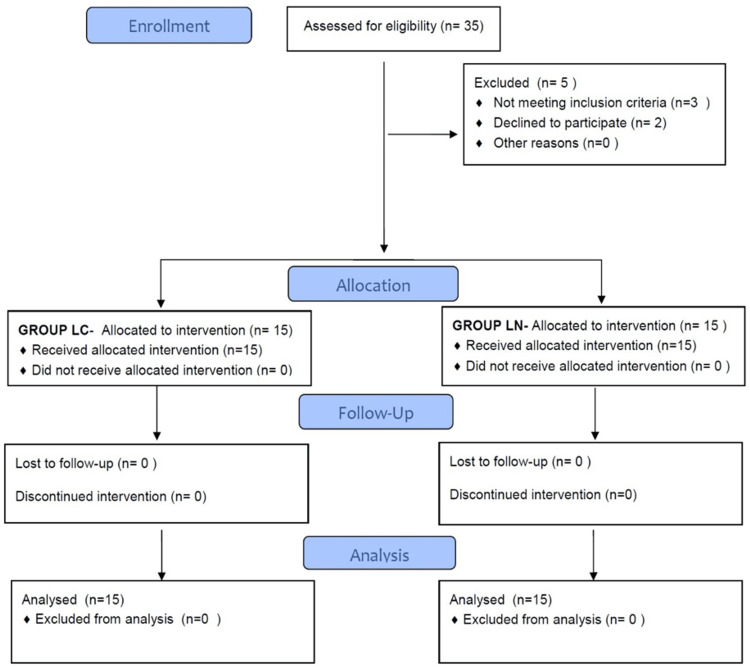
Consort flow diagram

All 30 cases were then analysed as shown in the flow diagram, Figure 1.

The demographic profile of patients is given in Table 1 and was comparable.

**Table 1:** Demographic profile of patients Yrs.: year; kg: kilogram; cm: centimetre; min: minutes. Values are expressed as Mean ± SD; p <0.05 is considered statistically significant.

Parameter	Group LC (n=15)	Group LN (n=15)	p-value	Fisher’s Test (x^2^)	Chi-Squared Test (x^2^)	Student’s T-test (t)
Age (Yrs.)	27.93 ± 10.97	26.87 ± 8.45	0.900	1.33	-	-
Gender (numbers) Male: Female	14:1	15 0	1.000	1.034	-	-
Weight (kg)	65.80 ± 5.29	67.07 ± 5.31	0.518	-	-	-0.654
Height (cm)	166.53 ± 3.27	168.13 ± 3.48	0.205	-	-	-1.297
ASA PS (numbers) I: II	10:5	10:5	1.000	-	<0.001	-
Heart Rate (BPM) (Baseline)	90.27 ± 13.90	86.27 ± 9.17	0.361	-	-	0.930
Systolic BP (mmHg) (Baseline)	130.20 ± 10.44	126.20 ± 10.50	0.304	-	-	1.046
Diastolic BP (mmHg) (Baseline)	77.53 ± 7.89	80.87 ± 5.85	0.200	-	-	-1.314
Duration of surgery	134.33 ± 32.4	134.07 ± 40.9	0.984	-	-	-

Intraoperative and postoperative hemodynamic parameters (HR, SBP and DBP) were comparable in both the groups at all the corresponding time points, LC and LN, except in DBP at 90 mins, where in group LC the mean was 77.00 ±6.32 min and in group LN it was 81.71 ±6.25 min, which was statistically significant (p=0.054)

Analgesic efficacy

**Figure 2:**
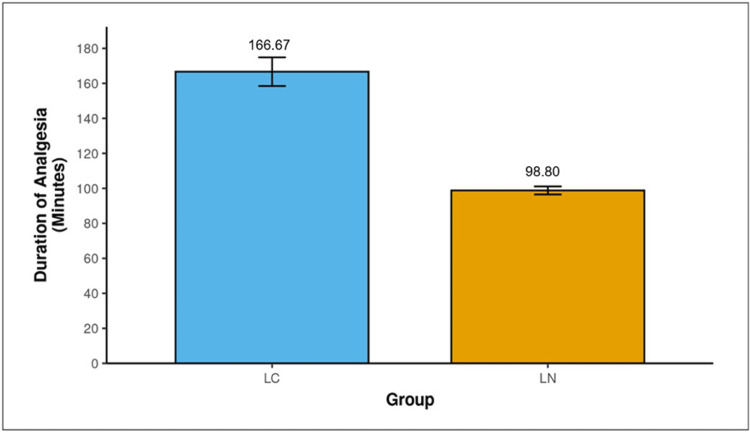
Comparison of duration of analgesia in LC and LN groups

The mean duration of effective analgesia was 166.67 ±31.72 min in group LC and 98.80 ±8.99 min in group LN (p <0.001), and the difference was statistically significant (Figure 2, Table 2).

**Table 2:** Characteristics of sensory block Values are expressed as Mean ± SD and as Median (IQR); p <0.05 is considered statistically significant, min =minutes.

Parameters	Group LC (n=15)	Group LN (n=15)	p-value	Wilcoxon -Mann-Whitney U Test (W)	Student’s T-test (t)
Duration of analgesia (minutes)	166.67 ± 31.72	98.80 ± 8.99	<0.001		7.973
Time to T10 (onset of sensory block) (min)	6.53 ± 1.60	4.00 ± 1.69	0.001	190.00	-
Time to achieve maximum height of sensory block (min)	11.53 ± 3.72	6.67 ± 1.45	<0.001	216.500	-
Max sensory block height	T6 [T6-T8]	T6 [T6-T8]	0.591	100.00	-

Sensory block characteristics

**Figure 3:**
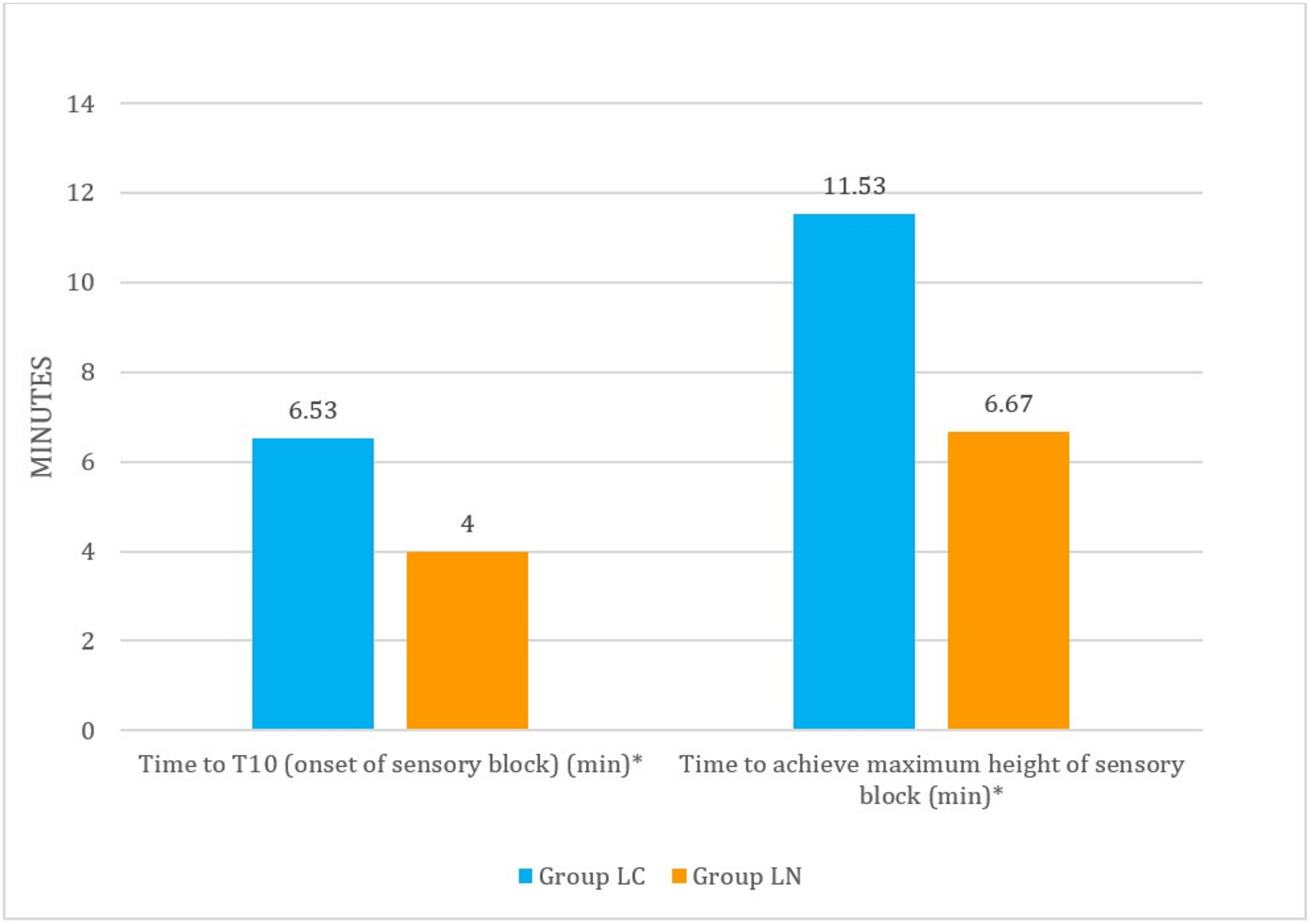
Comparison of time of onset and time to achieve maximum sensory block in LC and LN groups

The mean time of onset of sensory block was longer in the LC group, 6.53 ±1.60 min, as compared to the LN group, 4.00 ±1.69 min (p =0.001), which was statistically significant. The mean time to achieve a maximum height of the sensory block was 11.53 ±3.72 min in group LC and 6.67 ±1.45 min in group LN, and the difference was statistically significant (p <0.001). The maximum height of sensory block achieved was comparable in both groups (Table 2, Figure 3).

Motor block characteristics

All 15 patients in each group achieved complete motor block with a Bromage score of 3.

The maximum motor block (maximum motor block achieved within the first 20 mins) was achieved, and the motor block at the time of rescue analgesia was comparable in both groups (Table 3).

**Table 3:** Motor block at the time of rescue analgesia Values are expressed in numbers(percentage), p <0.05 is considered statistically significant.

Motor Block at the Time of Rescue Analgesia (Bromage Score)	Group LC (n=15) No. of pts (%)	Group LN (n=15) No. of pts (%)	p-value	Fischer Exact Test (x^2^)
0	9 (60.0%)	7 (46.7%)	0.465	3.050
1	4 (26.7%)	4 (26.7%)		
2	1 (6.7%)	4 (26.7%)		
3	1 (6.7%)	0 (0.0%)		

Side effects

None of the patients had any sedation in either of the groups, and all the patients were easily arousable. No major side effects were observed in either of the groups, except one patient (6.7%) in group LC and one patient (6.7%) in group LN, who developed nausea, which was managed with injection Ondansetron 4 mg intravenous. Five patients (33.3%) in group LC and three patients (20.0%) in group LN had shivering, which was managed promptly by injection of Tramadol 25mg.

Pain score

A meaningful comparison of pain scores in the postoperative period was not possible since 7/15 (46.6%) patients required conversion to GA for the conduct of the procedure in the levobupivacaine alone group and were given alternative analgesics. Hence, the pain score in the postoperative period for group LN was not counted.

## Discussion

One of the most frequently performed surgical procedures for the lower limb is for femoral fracture fixation. Subarachnoid block is a common anaesthetic technique to facilitate these surgical procedures. We studied 30 patients who were scheduled for femoral fracture surgery. They were randomly allocated to receive clonidine or normal saline as an adjuvant with hyperbaric levobupivacaine. The key findings were that the group receiving the levobupivacaine-clonidine combination had a longer duration of analgesia than the levobupivacaine-only group. The onset of block and time to achieve the maximum height of sensory block were significantly delayed in the levobupivacaine-clonidine group; however, the maximum height of sensory block and the maximum motor block achieved were comparable. There was no incidence of hemodynamic instability in either of the groups. A few incidences of nausea and shivering were observed with similar frequency in both groups.

Recently, levobupivacaine and ropivacaine have found a place for themselves due to their safety when compared to racemic bupivacaine. As per a previous report, it has been found that the equipotency ratio of levobupivacaine and racemic bupivacaine is 1:1. Usually, a volume of 3.0-3.5 ml is sufficient for the conduct of orthopaedic lower limb procedures [8-11]. Hence, we decided to choose 3.0 ml of hyperbaric levobupivacaine (0.5%) for our patients. In our study, we found that this dose and volume of drug were sufficient to initiate adequate blocks for all our patients. However, the duration of effective analgesia was shorter than the duration of the surgical procedure in the patients who received levobupivacaine only. These patients required conversion to GA for the completion of the surgical procedure. So, 15 mg of levobupivacaine alone was not found to be adequate for the conduct of femur surgeries.

With the addition of 15 mcg clonidine, the duration of effective block increased, which reinforces the previous knowledge that adjuvants prolong the duration of subarachnoid block. Clonidine acts on the alpha-2 receptors, which are present centrally and peripherally. When given intrathecally, it acts on the postjunctional alpha-2 receptors in the dorsal horn of the spinal cord and inhibits the secretion of substance P [12-14].

Our results regarding the primary outcome, i.e., duration of effective analgesia, are consistent with the previous findings. In our study, the duration of effective analgesia in the levobupivacaine-clonidine group was longer than that in the levobupivacaine with normal saline group, and the difference was statistically significant (p <0.001). Similar findings were observed in a study done by Krishna et al., where the mean duration of analgesia was 251.72 ±31.62 min in the levobupivacaine alone group, 294.68 ±43.35 min in the levobupivacaine-clonidine (15 mcg) group, and 355.76 ±52.19 min in the levobupivacaine-clonidine (30 mcg) group [7]. There was a statistically significant difference (p <0.001) among the three groups regarding the duration of analgesia. Other studies have also shown the dose-dependent increase in the duration of analgesia [5,7,11,15-17]. We also did not observe sedation in any of our groups, though one would expect some sedation due to the addition of clonidine. However, previous research also validates our findings, as lower doses of 15-30 mcg of clonidine have not been associated with sedation [5]. Sedation was observed in studies that used a higher dose of intrathecal clonidine, 50-75 mcg.

In our study, the mean time for onset of sensory block (time to T10) was longer ( 6.53 ±1.60 min) in the levobupivacaine-clonidine group compared to the (4.00 ±1.69 min) levobupivacaine-only group, and the time to achieve maximum block height was also longer in the LC group, 11.53 ±3.72 min. Like our findings, Kakunje et al. also found that the onset of sensory block was delayed with the addition of clonidine, which was 8.27 ±1.36 min and 7.40 ±2.11 min without clonidine, but the time to maximum block height was comparable [5]. However, in the study by Agarwal et al., no difference was observed with respect to both onset and time to achieve maximum sensory level in the combination group compared to only the levobupivacaine group [16]. This difference may be due to the low dose of local anaesthetic used in their study [16].

In our study, we found that the median block height achieved was T6 [T6-T8] in both groups. In a number of previous studies using the isobaric solution of levobupivacaine, they found that the drug had a very unpredictable spread [8,9,18]. Sanansilp et al. showed that the isobaric formulation is associated with a wide range of peak levels from C8 to L1 compared to the hyperbaric formulation, which was T2 to T7 [18]. The isobaric drug tends to give a very unpredictable block spread, but with the hyperbaric solution, the drug effect becomes more predictable and hence may be the preferred type of drug formulation for lower limb procedures.

All the patients in both groups in our study attained adequate motor blockade before the beginning of surgery.

In our study, we used 15 mcg of intrathecal clonidine as an adjuvant. Consequently, we observed that with this dose, the hemodynamics were stable, and none of our patients developed bradycardia or hypotension. Likewise, Krishna et al. also found no significant changes in hemodynamic parameters between the control and clonidine groups [7]. In contrast to our study, Agarwal et al. reported a significant fall in mean systolic blood pressure immediately after spinal with 30 mcg clonidine with 9 mg hyperbaric bupivacaine, but they found no significant hemodynamic fluctuations with 15 mcg clonidine [16]. Consequently, it may be deduced that the hemodynamic profile is preserved when clonidine at lower dosages, 15 mcg, is used. Clonidine, unlike other adjuvants, e.g., opioids, does not cause pruritus and other psychiatric issues [19].

We found that the group receiving only levobupivacaine had insufficient duration of block, so most of these patients 85.7% required intraoperative supplemental sedation and analgesia. However, with the addition of clonidine, the duration of effective analgesia increased, and consequently, in the levobupivacaine-clonidine combination group, 26.7% patients required supplemental analgesia during the surgery. Similar findings regarding the duration of block have been noted with a hyperbaric solution of levobupivacaine without the addition of adjuvants in a previous study, where the effect was shown to fade within one hour [18].In a recent study using the hyperbaric solution, the two-segment regression time was noted to be 130 ±20 min with 50 mcg clonidine [20]. These results are in agreement with our findings on the duration of the block. Thus, when using the hyperbaric solution, due consideration should be given to the expected duration of the procedure while deciding the dose of the drug, and adjuvants should be added to prolong the duration of effective analgesia. In a study done by Testa et al. in 2019, the average duration of surgery for intramedullary nailing for the shaft of femur fracture was 79.7+-21.7 min (range 45-130 min), while in our study, the average duration of surgery was 134.33 ± 32.4 min in the group LC and 134.07 ± 40.9 min in the LN group [21]. In our institute, most of these surgeries are done by trainee residents, thus resulting in prolonged surgical duration outlasting the duration of analgesia in many of our cases.

Strengths of our study are that it is one of the very few studies that have compared clonidine as an adjuvant to hyperbaric levobupivacaine in lower limb surgeries. We have recorded sensory and motor block characteristics, including the onset of sensory block, time to achieve maximum height of sensory block, maximum height of sensory block, duration of effective analgesia, and maximum motor blockade achieved. We conducted this study on a uniform group of patients, i.e., patients undergoing surgery for a femoral fracture requiring intramedullary nailing.

Limitations

This is a single-centric study with a small sample size due to the limitations of time and resources. Multicentric studies with a large sample size can throw more light on the use of clonidine with levobupivacaine in subarachnoid block.

## Conclusions

Based on the above observations, we conclude that the addition of 15 mcg of clonidine to 15 mg of 0.5% hyperbaric levobupivacaine provides a longer duration of effective analgesia with minimal side effects.

We recommend the use of 15 mcg clonidine as an additive to 15 mg of 0.5% hyperbaric levobupivacaine for femoral fracture fixation under a subarachnoid block, which provides safe and longer duration of effective analgesia without notable hemodynamic changes and with minimal or no side effects.
